# Dysregulation of Host–Pathogen Interactions in Sepsis: Host-Related Factors

**DOI:** 10.1055/s-0044-1787554

**Published:** 2024-07-01

**Authors:** Sebastiaan C.M. Joosten, Willem J. Wiersinga, Tom van der Poll

**Affiliations:** 1 Centre for Experimental and Molecular Medicine, Amsterdam University Medical Center, Amsterdam, The Netherlands; 2 Division of Infectious Diseases, Amsterdam University Medical Center, Amsterdam, The Netherlands

**Keywords:** sepsis, review, pathophysiology, immune dysregulation, host response

## Abstract

Sepsis stands as a prominent contributor to sickness and death on a global scale. The most current consensus definition characterizes sepsis as a life-threatening organ dysfunction stemming from an imbalanced host response to infection. This definition does not capture the intricate array of immune processes at play in sepsis, marked by simultaneous states of heightened inflammation and immune suppression. This overview delves into the immune-related processes of sepsis, elaborating about mechanisms involved in hyperinflammation and immune suppression. Moreover, we discuss stratification of patients with sepsis based on their immune profiles and how this could impact future sepsis management.

Sepsis, a life-threatening condition resulting from the body's unbalanced response to infection, remains a significant challenge in modern medicine. Characterized by a dysregulated host response, sepsis can lead to multiorgan dysfunction and, in severe cases, death. Despite advances in our understanding of its pathophysiology, sepsis continues to be a leading cause of mortality worldwide. 1

The host response to sepsis is complex, involving a delicate interplay between a variety of immunological systems. While the initial response aims to contain and eliminate the invading pathogen, an excessive or prolonged reaction can result in collateral tissue damage and organ failure. This article delves into the intricacies of the sepsis host response, exploring the molecular and cellular mechanisms that underpin this destructive force.

## Initiation of the Host Response

Sepsis emerges when the sophisticated defense mechanisms, designed to protect our body from virulent organisms, become compromised. Rather than acting as our shield, they malfunction, setting off a chain of events that lead to organ failure. 2 This process starts with the introduction of the pathogen to the host. At this juncture, our immune recognition machinery is activated by a plethora of receptors termed pattern recognition receptors (PRRs). 3 These receptors play a key role in detecting a wide array of motifs from pathogens and damaged cells, known as pathogen-associated molecular patterns (PAMPs) and damage-associated molecular patterns (DAMPs), respectively. PRRs are a pivotal part of the innate immune system and are expressed in immunological and parenchymal cells. 4 The most well-known PRRs belong to the family of Toll-like receptors (TLRs). These receptors were initially discovered as the drivers of fruit fly embryogenesis in 1985, 5 and in the years since been found to be essential for pathogen recognition in humans as well. 6 TLRs have the capacity to identify an array of pathogenic motifs, attributable to their presence both inside and outside the cell. These motifs encompass bacterial cell wall components of both Gram-positive and Gram-negative bacteria, as well as viral DNA or RNA. 6 Since their initial discovery, the repertoire of PRRs has significantly expanded to include c-type lectin receptors, nucleotide oligomerization domain-like receptors, RIG-1-like receptors, and several cytosolic DNA sensors. Upon binding to their respective ligands, these receptors initiate a variety of intracellular signaling pathways. This activation subsequently triggers various transcription factors, including nuclear factor kappa-light-chain-enhancer of activated B cells (NF-κB), cAMP response element-binding protein (CREB), activator protein 1 (AP-1), and subsequent production of a range of proinflammatory cytokines, thereby initiating the innate immune response. 6

If patients cannot effectively combat the initially localized infection, PRRs remain persistently activated, resulting in excessive pro- and anti-inflammatory activity. 7 Given their central role in the development of sepsis, modulating PRR signaling is viewed as a promising therapeutic approach. Specifically, the modification of TLR4 signaling pathway has previously gained attention because of its ability to detect lipopolysaccharides (LPS) on Gam-negative bacteria, as well as several DAMPs. 2 4 6 7 8 Despite promising preclinical outcomes, the phase 3 trial of the TLR4/MD2 inhibitor eritoran in patients with sepsis did not yield any clinical benefit. 9 The receptor for advanced glycosylation end products (RAGE) and the triggering receptor expressed on myeloid cells-1 (TREM-1) are two additional PRRs that have been the subject of research. RAGE interacts with various DAMPs, such as high mobility group box 1 (HMBG1), S100A8/9, and MAC-1 (a heterodimer of CD11b and CD18), in addition to advanced glycosylation end products (AGEs). 10 Elevated levels of AGEs have been observed in the heart and brain of sepsis patients, potentially initiating inflammation in these areas. 11 Recent preclinical studies have highlighted the possible benefits of inhibiting the interaction between RAGE and a key molecule in its signaling pathway. 11 In sepsis, TREM-1 is abundantly expressed on monocytes and neutrophils. Functionally, TREM-1 synergizes with TLRs to mediate the release of proinflammatory cytokines in response to a spectrum of PAMPs. 12 Nangibotide, a TREM-1 inhibitor, was recently investigated in the ASTONISH phase 2 trial. Despite failing to meet its primary endpoint of significant improvement in Sequential Organ Failure Assessment (SOFA) score at a predetermined soluble TREM-1 value (>400 pg/mL), the study found that nangibotide may decrease SOFA scores in patients admitted to the intensive care unit with higher levels of sTREM-1. 13

## Escalation of Inflammation

Persistent inflammation is central to the host's dysregulated immune response in sepsis. The sustained exposure to DAMPs and PAMPs leads to leukocyte influx, endothelial activation, and the engagement of the complement system. At the onset of sepsis, there is a steep increase in the production and release of acute-phase proteins and cytokines that further propagate the immunological reaction. Tumor necrosis factor (TNF) and interleukin (IL)-1β are notable proinflammatory cytokines, whereas IL-6 exhibits both pro- and anti-inflammatory properties. 14 IL-17, originating from T helper and innate lymphoid cells, further amplifies the production of TNF and IL-1β. 15 HMGB1, which serves as both a cytokine and a DAMP, is produced at elevated levels during sepsis. 8 Its capacity to activate multiple PRRs made it an appealing therapeutic target; however, its evaluation has not extended beyond the preclinical stage. 4 Collectively, these and other interactions between PRRs and PAMPs/DAMPs along with a plethora of secreted inflammatory mediators contribute to establishing a complex and heterogeneous proinflammatory environment in patients with sepsis.

Neutrophils serve as the frontline defense force of the immune system. This role makes them an integral part of protective immunity, yet their comprehensive inflammatory arsenal can also turn against the host. Once an infection is detected in the body, neutrophils rapidly migrate to the site of infection and unleash their destructive potential. Their granules carry proteosomes, lysosomes, and radical oxygen species which are released once they encounter the infected tissue. This creates an area enriched with PAMPs and DAMPs, facilitating activation of the surrounding leukocytes. 16 Traditionally, neutrophils have not been primarily associated with the function of antigen presentation. However, a recent study has identified a subset of aged (CXCR4 + ) neutrophils that proficiently presents antigens to T cells, which in turn induces interferon-γ release. 17 Furthermore, neutrophils are capable of forming neutrophil extracellular traps (NETs). These extracellular matrices, composed of cell-free DNA, histones, myeloperoxidase, and elastase, facilitate pathogen neutralization, and enhance phagocytic activity. NETs are secreted in response to diverse stimuli, including bacteria, fungi, viruses, parasites, and even neoplastic cells. 18 19 NET release can occur self destructively or vitally, after which the neutrophil remains functionally active. Host factors have been shown to play a role in NET formation. For example, neutrophils from diabetic patients more readily undergo NETosis when compared with their euglycemic counterparts. 20 Additionally, neutrophil heterogeneity also affects NETosis; for instance, aged neutrophils have been reported to be more prone to NET formation. 21 Interestingly, macrophages also possess the ability to produce extracellular traps; however their role in sepsis has not been elucidated. 22 The presence of NETs can also be detrimental to the host, being highly proinflammatory containing vast amounts of histones and DNA, both potent DAMPs. Indeed, NETs can cause tissue damage, vascular leakage, and intravascular thrombosis. 18 23

The complement system represents a crucial component of the innate immune response. 24 This system can be initiated via three distinct pathways in response to an infection. First, the lectin pathway is activated when mannose-binding lectin binds to carbohydrates present on the surface of pathogens. Second, the classical pathway is initiated upon the binding of Complement C1 to surface-bound antibodies. Lastly, the alternative pathway commences when hydrolyzed C3 associates with microbial surfaces, subsequently acting as an amplification mechanism to propagate the complement cascade. When these pathways are activated, the complement system facilitates the release of potent chemotactic agents, namely C3a and C5a, leading to the mobilization of leukocytes through increased vascular flow, permeability, and adhesion. Simultaneously, the complement system facilitates the formation of the terminal complement complex, forcing bacterial lysis. 6 In the context of sepsis, there is pronounced activation of the complement system, making it a primary contributor to hyperinflammation. 25 While complement activation is acknowledged as a common occurrence in sepsis, a recent study found no correlation between complement usage and other inflammatory markers in severe sepsis cases. 26 This could indicate that, in patients with advanced sepsis, the complement system is already maximally activated, rendering further proinflammatory triggers ineffective in enhancing complement activity. 26 Previous studies have shown that increased levels of C5a correspond to poorer clinical outcomes in sepsis. 27 Given the influence of complement activation in sepsis, there is growing enthusiasm for complement-targeted therapeutic approaches. Blocking C5a has shown success in the preclinical setting; however, no therapies have reached clinical use in sepsis. 28

The innate responses mentioned above are consistently associated with disruptions in the coagulation system, which is a widely observed phenomenon in sepsis. 29 The spectrum varies from minor activation of coagulation to disseminated intravascular coagulation (DIC), which is accompanied by impaired hemostasis due to the depletion of clotting factors and platelets. 30 Notably, up to a third of sepsis patients meet the diagnostic criteria for DIC. 31 The coagulopathy observed in sepsis can be attributed to a complex interplay of factors including the formation of thrombin, the impairment of anticoagulant mechanisms, and a reduction in fibrin removal. These disturbances in coagulation largely result from the intricate interaction between coagulative and inflammatory pathways. Tissue factor (TF) serves as a principal catalyst of the procoagulant state, initiating the coagulation cascade through the formation of complexes with clotting factor VIIa. 29 Beyond its expression in the perivascular environment, TF is also expressed on monocytes and macrophages, with expression levels rising upon activation. 32 Once TF is inhibited, thrombin formation is prevented in nonhuman primate sepsis models demonstrating the importance of this extrinsic pathway. 33 In the complex procoagulant and hyperinflammatory environment in sepsis, platelet activation plays a central role by propagating both coagulation and inflammation. 34

Besides the increase of thrombin formation, there is also a distinct lack of anticoagulant measures counteracting hemostasis. Three essential proteins serve this purpose: tissue factor pathway inhibitor (TFPI), antithrombin, and activated protein C (APC). TFPI and antithrombin both poorly function in sepsis, largely due to the disturbed production of glycosaminoglycans on the endothelial surface to which TFPI is attached and which facilitates the function of antithrombin. APC performs an important antithrombotic function by proteolytically inhibiting clotting factors Va and VIIIa. Its function in sepsis is impaired because of reduced production of its precursor protein C by the liver, increased consumption, and a decrease in its activator thrombomodulin. 29 In addition to the diminished functionality of these factors, fibrinolysis is compromised by elevated levels of plasminogen activator inhibitor type 1, which hinders plasmin generation and thereby the capacity to degrade fibrin matrices. 35

**Fig. 1:**
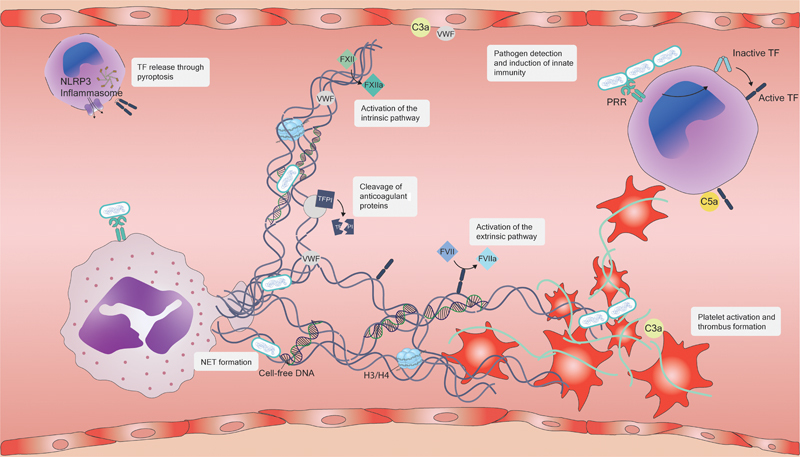
Immunothrombosis within the microvasculature in sepsis. Interactions between the immune and coagulation systems culminate in the formation of microvascular thrombi during sepsis, promoting local inflammation. Activation of leukocytes via their PRRs induces the surface expression of TF, catalyzing the enzymatic initiation of the extrinsic coagulation pathway. Concurrently, assembly of the NLRP3 inflammasome triggers pyroptosis, facilitating the extracellular release of TF. NETs, produced by activated neutrophils, not only contain immunogenic cell-free DNA and histones but also interact with VWF and platelets, thereby initiating primary hemostasis. Additionally, enzymes within NETs, such as myeloperoxidase, degrade anticoagulant proteins like TFPI and TM. Lastly, the binding of TF and the negative charge of NETs activate the extrinsic and intrinsic coagulation pathways, respectively. Complement components upregulate TF on leukocytes and VWF on endothelial cells. Reciprocally, these components also activate coagulation factors and platelets, thus propagating both coagulation and the inflammatory response. C3a, complement component 3a; FXIII, factor XIII; H3/4, histone 3/4; NET, neutrophil extracellular trap; NLRP3, NLR family pyrin domain containing 3; PRR, pattern recognition receptor; TF, tissue factor; TFPI, tissue factor pathway inhibitor; TM, thrombomodulin; VWF, von Willebrand factor.

Immunothrombosis is a term that describes a complex physiological process where the immune system and the coagulation system interact in a coordinated manner ( Fig. 1 ). This interplay between fibrin, neutrophils, monocytes, and platelets has bactericidal effects by ensnaring pathogens, thereby localizing the infection, suppressing tissue invasion and dissemination. 36 However, when left unchecked, microvascular thrombosis can lead to local hypoxia and eventually organ failure. NETs effectively stimulate the coagulation cascade via both the contact and extrinsic pathways, owing to their negative charge that activates factor XII and their affinity for binding to TF. Furthermore, NETs harbor proteolytic enzymes capable of cleaving antithrombotic proteins, including TFPI and thrombomodulin. 37 Additionally, cell-free DNA and histones, such as H3 and H4, present in NETs serve as potent activators of platelets, thereby promoting fibrinogenesis. 38 These activated platelets reciprocally stimulate surrounding neutrophils, leading to the further generation of NETs. 34 38

The complement and coagulation systems not only exhibit functional parallels but also operate in a synergistic manner. Complement factors stimulate the expression of TF on leukocytes, activate platelets, and trigger the release of von Willebrand factor from endothelial cells. 39 The physiological implications of these interactions were demonstrated in a preclinical primate model of *Escherichia coli* -induced sepsis, where administration of the C3 inhibitor Compstatin resulted in attenuated manifestations of microvascular thrombosis. 40 Pyroptosis, a form of inflammatory cell death, has recently been implicated in the modulation of immunothrombosis. Pyroptosis is predominantly observed in nonmyeloid-derived leukocytes and initiated by the activation of PRRs. Such activation triggers the assembly of inflammasomes, leading to the subsequent cleavage of Gasdermin D. These cleavage products then form pores in the cellular membrane, facilitating the release of a myriad of inflammatory cytokines and DAMPs as well as TF. 41

## Anti-inflammatory Mechanisms and Immune Suppression

Much like other physiological systems, the immune system has built-in mechanisms to control overactivation and reduce its harmful effects. In the context of sepsis, these regulatory mechanisms may not only fail to adequately constrain tissue damage but also induce a state of immunosuppression, rendering the host vulnerable to secondary infections. 42 43 44 One potential contributor to the immunosuppression in sepsis is the depletion of lymphogenic effector cells. Lymphopenia is a prevalent characteristic in sepsis, manifesting in more than half of sepsis patients. 45 This reduction is largely ascribed to the T cell compartment, with natural killer (NK) and B cells contributing to a lesser extent. 46 Postmortem analyses of patients who succumbed to sepsis revealed the presence of cellular apoptosis in various organs, implicating it as a primary driver of lymphopenia. 47 Concurrently, elevated lymphocyte trafficking to infected tissues and diminished output from the thymus and bone marrow may also contribute to reduced lymphocyte counts. 44 Although lymphopenia has been consistently associated with negative clinical outcomes and elevated mortality rates, its role as either a causal factor or an epiphenomenon concurrent with disease progression remains an area warranting further investigation. The improved survival rates observed when inhibiting various apoptotic pathways in preclinical sepsis models support the pathophysiological importance of lymphocyte dysfunction in sepsis. 48 In addition to the reduction in lymphocyte counts, the residual lymphocytes in sepsis patients often exhibit markers of cellular exhaustion. This compromised state not only impairs their ability to activate adjacent cells but also curtails cytokine production and heightens their vulnerability to apoptosis. 49 Contributing to this state of exhaustion are checkpoint regulators, membrane-bound proteins that modulate downstream signaling from the T cell receptor. Among these are inhibitory molecules such as cytotoxic T-lymphocyte-associated protein 4 (CTLA-4), T cell immunoglobulin mucin domain containing-3 (TIM-3), and programmed death receptor 1 (PD-1). These inhibitory markers are commonly found on circulating T cells in sepsis and are associated with lymphopenia and increased mortality rates. 50 The ligands for PD-1, namely PD-L-1/2, are expressed on the surface of antigen-presenting cells and exhibit increased expression in sepsis as well. While PD-1 inhibition has proven effective in reinvigorating T cell responses within oncology, its application in sepsis has shown promise in terms of tolerability and some effectiveness but has not yet advanced past phase 2 clinical trials. 51 52

A defining feature of sepsis is the impaired function of monocytes and macrophages. When exposed to additional bacterial antigens, monocytes derived from sepsis patients exhibit reduced production of proinflammatory cytokines such as TNF and IL-1β, whereas their anti-inflammatory responses are either preserved or even heightened. This condition has been referred to as “endotoxin tolerance,” 53 albeit the term monocyte/macrophage “reprogramming” better captures the modified responsiveness of these cells. Additionally, human leukocyte antigen-DR isotype (HLA-DR) expression on monocytes is decreased in sepsis, compromising their ability to present antigens to T cells. Diminished expression of HLA-DR on peripheral monocytes serves as a predictive marker for adverse outcomes in sepsis and overall immunosuppression. 54 Endotoxin tolerant monocytes have likely been epigenetically reprogrammed through DNA methylation and histone modification, which in turn reduces their capacity for NF-κB activation. 2 55 Notably, this trait appears to be more prevalent in short-lived macrophages and monocytes, as opposed to resident macrophages in the liver and lungs, which exhibit signs of immune priming and display inflammatory responses upon reactivation. 56

The balance between anti-inflammatory and proinflammatory cells is a critical factor in the emergence of immunosuppression. Regulatory T cells (Tregs), identified by the presence of the Foxp3 transcription factor, act as the immune system's arbiters of cellular responses. These cells are commonly found in immune-privileged sites like the central nervous system and eyes. 6 During sepsis, Tregs are notably more prevalent and contribute to the suppression of adjacent leukocytes, further exacerbating the immunosuppressive state. 57 Evidence also suggests a beneficial role for these cells in sepsis as higher levels of Foxp3 have been observed in sepsis survivors compared with nonsurvivors, suggesting that their presence could also be advantageous to the host. 58 Amid infectious conditions, the bone marrow expedites the production and release of immature myeloid cells via a process termed “emergency myelopoiesis.” Intriguingly, a portion of these cells are immunosuppressive, known as myeloid-derived suppressor cells (MDSCs). These cells are relatively rare in healthy subjects but become more abundant in patients with sepsis. 59 Single-cell RNA sequencing of peripheral blood mononuclear cells has identified a unique monocyte gene signature, termed MS1, that is indicative of an immunosuppressive monocyte state specific to sepsis. 60 When isolated, these cells demonstrated the ability to inhibit T cell proliferation and activation, suggesting they may belong to the MDSC subset. 60 Single-cell RNA sequencing of whole blood identified another immunosuppressive signature derived from immature neutrophils, which shared many similarities with MS1 cells, pointing to parallel processes underlying their generation. 61 Notably, the expression of IL1R2, a receptor that serves to downregulate IL-1 stimulation, was elevated in both cell types. The noticeable presence of both cell types in sepsis, coupled with their association with adverse outcomes, underscores the critical role of myeloid cell-derived immune suppression in the pathophysiology of sepsis.

In the past decade, there has been a profound advancement in our understanding of how cellular metabolism influences the functional capabilities of immune cells. Under quiescent conditions, immune cells predominantly rely on oxidative phosphorylation (OXPHOS) for their energy needs, a process that is highly efficient and generates ample adenine triphosphate (ATP) through the tricarboxylic acid (TCA) cycle and electron transport chain. 62 However, during infection, the energy requirements rise, necessitating a swift augmentation in ATP production. To meet this demand, cells elevate their glucose uptake and initiate aerobic glycolysis, a phenomenon termed “the Warburg effect.” 63 In addition to rapid ATP production, this metabolic shift also yields other byproducts such as nicotinamide adenine dinucleotide phosphate (NADPH), which can be utilized for lipid biosynthesis or protein assembly. In neutrophils, enhanced glycolysis is crucial for the generation of NETs. 64 Extensive research on macrophages and dendritic cells has identified multiple mechanisms through which metabolic reprogramming occurs. A principal mechanism centers on the elevated expression of hypoxia-inducible factor (HIF)-1α, which in turn facilitates glucose uptake through glucose transporter 1 (GLUT1) and enhances glycolysis via lactate dehydrogenase. 65 Alternative approaches include the stimulation of the mammalian target of rapamycin (mTOR) pathway and the inhibition of AMP-dependent protein kinase, which collectively encourage protein synthesis while limiting fatty acid biosynthesis. 66 Conversely, immunosuppressive states necessitate the downregulation of HIF-1α and the upregulation of fatty acid transporters like CD36 and carnitine palmitoyl transferase 1 (CPT1). This facilitates the entry of fatty acids into the mitochondria for β-oxidation, thereby promoting OXPHOS. 63 This metabolic switch between OXPHOS and glycolysis is orchestrated by NAD + -dependent deacetylases, primarily sirtuin 1 and 6, which serve as key sensors of energy availability. 67 Interestingly, glycolytic conditions can also lead to immunosuppression through the production of lactate, which can inhibit cytokine production through the TLR/NF-κB pathway and increase the expression of anti-inflammatory genes like *IL10* in macrophages. 68 69 70

Metabolic plasticity allows for the smooth transition between metabolic states and is paramount to the function of immune cells. Under septic conditions, extended interaction with antigens and aberrant modulation of the inflammatory cascade culminate in the loss of this plasticity and transition to a state of “immunometabolic paralysis.” 71 Monocytes isolated from sepsis patients exhibiting an immunotolerant phenotype demonstrate compromised utilization of both OXPHOS and glycolysis for sufficient ATP synthesis. This impairment is plausibly attributed to anomalies in mTOR signaling and mitochondrial functionality. 72 Analogous metabolic perturbations have been observed in T cells derived from sepsis patients. In vitro studies have demonstrated that administering IL-7 to T cells from these patients can ameliorate certain indicators of metabolic failure via the activation of mTOR. 73 The observed immunometabolic dysregulation in sepsis contributes significantly to overall complexity of the disease and is posited to be a key determinant in the overarching immunosuppressive state.

## Trained Immunity

The adaptive immune system is renowned for its ability to generate a highly specialized response to pathogens through a range of cellular processes that confer pathogen specificity to adaptive immune cells. 6 Historically, the innate immune system has not been associated with such intricate modifiable machinery. However, emerging evidence indicates that we may have done it a disservice by underestimating its adaptability. The concept of “trained immunity” is a relatively recent addition to immunological literature, detailing the ability of innate immune cells to acquire a form of immunological memory. When exposed to an immunogenic stimulus, these cells experience a priming phase that enhances their future reactivity, a change that can be sustained for up to a year. 74 This concept has garnered attention in the field of sepsis research, serving both as a framework to explain the heterogeneous immune responses among sepsis patients and as a potential therapeutic avenue for addressing immunoparalysis.

Trained immunity is predominantly linked to myeloid lineage immune cells, although evidence indicates that NK cells and innate lymphoid cells can also be trained. 75 Various immunological triggers can set this process in motion, ranging from whole pathogens like the weakened tuberculosis strain in the bacillus Calmette-Guérin (BCG) vaccine to pathogen components like β-glucans derived from the cell wall of fungi. 74 Notably, LPS is known for fostering endotoxin tolerance, yet it may also elicit trained immunity and amplify inflammation when administered at lower concentrations. 76 Endogenous stimuli, including host-derived proteins like hormones and lipoproteins, can likewise induce trained immunity. These inducers collectively contribute to the formation of immunological memory, albeit to varying degrees. 74

**Fig. 2:**
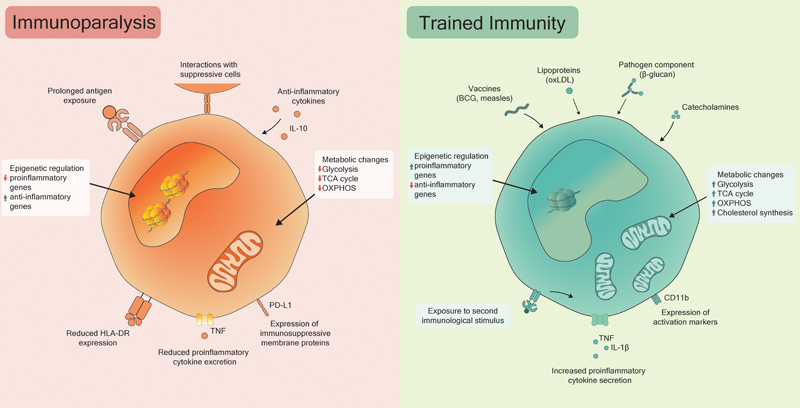
Mechanisms of immunoparalysis and trained immunity. Similar mechanisms underlie the phenomena of immunoparalysis and trained immunity. During sepsis, enduring inflammatory conditions create an environment rich in DAMPs and PAMPs, which continually activate TLRs. Coupled with anti-inflammatory conditions induced by the immune system, this results in cellular reprogramming in leukocytes, ultimately rendering them immunoparalyzed. Metabolic and epigenetic alterations orchestrate this reprogramming, leading to diminished energy availability and epigenetic repression of proinflammatory genes. Consequently, leukocytes show reduced responsiveness to subsequent immune stimuli, characterized by decreased expression of HLA-DR, elevated expression of anti-inflammatory receptors and ligands, and diminished cytokine production. Trained immunity is initiated when innate immune cells encounter immunological stimuli, priming them for enhanced future responses. Notable inducers include β-glucan and the BCG vaccine. Upon initial exposure, metabolic pathways are upregulated, augmenting cellular energy reserves. Histone modifications and DNA methylation prime the cell for rapid cytokine production upon receipt of a second stimulus, leading to heightened immune responsiveness upon reactivation. BCG, bacillus Calmette-Guérin; DAMPs, damage-associated molecular patterns; HLA-DR, human leukocyte antigen-DR isotype; IL-1β, interleukin 1β; IL-10, interleukin 10; oxLDL, oxidized low-density lipoprotein; OXPHOS, oxidative phosphorylation; PAMPs, pathogen-associated molecular patterns; PD-L1, programmed death-ligand 1; TCA, tricarboxylic acid cycle; TLRs, Toll-like receptors; TNF, tumor necrosis factor.

Analogous to the immunological reconfiguration observed in immune-suppressed cells, a synergy of epigenetic and metabolic modulations drives trained immunity. Metabolic alterations include the upregulation of both glycolysis and the TCA cycle, facilitating swift ATP generation and the production of TCA intermediates that are precursors for fatty acid and cholesterol synthesis—two pivotal pathways in trained immunity. 77 Intriguingly, despite the common downregulation of OXPHOS during inflammatory states, many agents that induce trained immunity result in elevated OXPHOS pathway activity. 78 This may offer an explanation for the long-lasting effects of trained immunity. Epigenetic modulation is intricately linked with metabolic changes ( Fig. 2 ). For instance, acetyl-CoA acts as an acetyl donor for histone acetyltransferases, whereas the TCA cycle metabolite fumarate can inhibit the histone demethylase KDM5. 79 While histone modification seems crucial in trained immunity, the contribution of DNA methylation is less clearly established. 74

The degree to which sepsis triggers trained immunity remains under active investigation; however, initial studies using animal models of sepsis have demonstrated signs of immunological training in hematopoietic precursor cells. 80 Sepsis may give rise to a heterogeneous population of leukocytes, exhibiting both trained and tolerant characteristics, and this composition could vary among patients.

In recent years, various studies have sought to leverage trained immunity for the prevention of secondary infections using the BCG vaccine. The ACTIVATE trial demonstrated some efficacy in reducing infections in elderly patients posthospital discharge. 81 However, in a recent trial BCG vaccination did not protect against clinically relevant respiratory tract infections in older adults 82 and in a large study aimed at preventing coronavirus disease 2019 (COVID-19) among health care workers through trained immunity, BCG vaccination did not reduce the incidence of viral infections. 83 Notably, these studies did not include patients recovering from sepsis, a group affected by immunoparalysis, thereby limiting the generalizability of the findings to this specific population. A recently published study introduces an innovative approach for eliciting trained immunity through the encapsulation of IL-4 in lipid nanoparticles. 84 Although traditionally linked with immune suppression and a shift toward type II immunity, IL-4 has demonstrated its ability to induce trained immunity. Administration of these nanoparticles successfully rejuvenated monocyte function in both mouse models of sepsis and cells harvested from healthy subjects intravenously injected with endotoxin. 84

## Stratification of Sepsis Patients Based on Their Host Response

Patients with sepsis do not express features of solely hyperinflammation or merely immunosuppression. Rather, the host response during sepsis is dysregulated in a mixed and heterogeneous way that shows strong variation between individual patients. Recently, several attempts have been made to identify subgroups of patients that are more alike based on large sets of host response characteristics. These approaches have also been applied retrospectively to datasets from previously conducted randomized trials in patients with sepsis, showing their potential value in selecting the right patients (i.e., those who are more likely to benefit) for selective immune modulatory therapies.

Much has been learned from investigations that used whole-blood leukocyte gene expression profiles to stratify patients with sepsis in more homogeneous subgroups using unsupervised clustering techniques. Two subgroups were identified in patients with sepsis caused by community-acquired pneumonia, named Sepsis Response Signatures SRS1 and SRS2. 85 Of these, SRS1 showed several features of immune suppression and was associated with a higher mortality. Importantly, in a retrospective analysis of a randomized trial studying the effect of corticosteroids in patients with septic shock, corticosteroids reduced survival of patients belonging to the SRS2 subgroup, while no treatment effect was detected in SRS1 patients. 86 The SRS stratification has been further developed into a quantitative SRS (SRSq) score (from 0 to 1), based on a 7- or 12-gene signature, that reflects the extent of immune dysfunction and is predictive of clinical outcomes in sepsis. 87 Blood transcriptomics was also used to come to different subgroups in other sepsis cohorts. Our group reported four subgroups (Mars1 to Mars4), in patients with sepsis, while another study reported three subgroups, termed “inflammopathic,” “adaptive,” and “coagulopathic”; further analyses of these subgroups revealed distinct dominant immunopathological pathways in each of them. 88 89 Collectively, these studies indicate that sepsis patients can be stratified into subgroups with more homogeneous host response characteristics based on their blood leukocyte transcriptomes, and that these subgroups have distinct clinical outcomes and possibly respond differently to specific sepsis therapies. Clearly, subgroups identified by unbiased clustering of blood transcriptome data differ across studies and consensus is needed in this regard.

In patients with acute respiratory distress syndrome, a common complication of sepsis, two subgroups named “hyperinflammatory” and “hypoinflammatory” have been identified based on clinical data and a limited set of plasma biomarkers. 90 These subgroups can also be identified in patients sepsis, and in a retrospective analysis of the PROWESS-SHOCK study, evaluating the effect of recombinant human APC in patients with septic shock, the response to this therapy differed by subgroup. 91

Together, these studies show that stratification of patients with sepsis based on host response features may not only provide insight into the risk of adverse outcomes, but also may assist in selecting subgroups of patients that are more likely to respond to sepsis therapies in a favorable way or—reversely—in avoiding certain therapies in those patients who are likely to be harmed by them. This approach, termed “predictive enrichment” of patient populations exposed to selected therapies, is key to advancement of precision medicine in sepsis.

## Conclusion

Pathogen recognition, hyperinflammation, immunothrombosis, and anti-inflammatory mechanisms all serve as pieces in the complex puzzle that characterizes the sepsis host response. Adding to this complexity is the patient- and pathogen-specific nature of how these components interact and affect the overall clinical picture. In light of this, it is not surprising that, despite an abundance of therapies showing promise in preclinical studies, none have made the leap to clinical practice.

A growing consensus among scientists calls for a direct confrontation of the difficult challenge presented by sepsis. 92 93 We must leverage our state-of-the-art computational and experimental techniques to formulate an integrated model of sepsis, one that can be customized according to the unique characteristics of the host and pathogen. This model would then serve as a foundation for the development of personalized therapeutic interventions for patients with sepsis.
